# Microbiome in Lower Urinary Tract Symptoms (LUTSs): Mapping the State of the Art with Bibliometric Analysis

**DOI:** 10.3390/life13020552

**Published:** 2023-02-16

**Authors:** Hadi Mostafaei, Hanieh Salehi-Pourmehr, Mohammad Sajjad Rahnama’i, Helia Mostafaei, Shahrokh F. Shariat, Sakineh Hajebrahimi

**Affiliations:** 1 Department of Urology, Comprehensive Cancer Center, Medical University of Vienna, 1090 Vienna, Austria; 2 Research Center for Evidence-Based Medicine, Iranian EBM Center, A Joanna Briggs Institute Center of Excellence, Tabriz University of Medical Sciences, Tabriz 5166-15731, Iran; 3 Department of Urology, St. Elisabeth-Tweesteden Hospital, 5022 Tilburg, The Netherlands

**Keywords:** microbiota, microbiome, functional urology, bibliometrics, scientometric study

## Abstract

Background: The main objective of this study is to provide the first characterization of the current research field of the clinical microbiome in LUTSs. Methods: First-of-its-kind scientometric insight into the historical development and structural state of the discipline is provided by a field analysis, mapping, and sub-analysis of articles for future research. On 22 December 2022, the entire Scopus database was searched without language or date restrictions. Search terms included “Chronic prostatitis”, OR “Interstitial cystitis”, OR “Lower urinary tract symptoms”, OR “Lower urinary tract dysfunction”, OR “Overactive bladder”, OR “Incontinence”, OR “Urolithiasis”, OR “Urothelium”, OR “Urine”, OR “Urology”, OR “urinary disorder”, OR “Pathophysiology”, OR “Benign prostatic hyperplasia”, OR “Benign prostatic enlargement”, AND “Microbiota”, OR “Microbiome”, OR “Urobio-ma”, OR “Urobiota; microflora”. The author and institutional data were transformed using the analytical tool Biblioshiny (a Shiny app for Bibliometrix), which took into account variations in author spelling as well as institutional naming and subgroups. Results: The specified search strategy was able to locate 529 documents from 267 sources published from 1981 to 2022. The average number of years from publication was 4.59 years. The authors with the most publications were Wolfe AJ and Brubaker I. The top three most collaborative networks were Loyola University Chicago, Loyola University Medical Center, and the University of California San Diego. The most frequently occurring words among the 50 nodes were: human, humans, nonhuman, female, adult, article, microbiology, microflora, microbiota, and controlled study. *Frontiers in Cellular and Infection Microbiology* and the *International Urogynecology Journal*, followed by *Nature Reviews Urology*, were the top three most relevant sources in microbiome research in urology. Conclusions: One of the most crucial requirements for developing research policies and anticipating the scientific requirements of researchers is paying attention to the evolution of various scientific fields. Understanding research gaps and future needs in microbiome research in urology can be effectively understood by paying attention to the models, maps, and visualizations used in this research, which are the results of systematic analysis of scientific products in the most esteemed scientific journals in the world.

## 1. Introduction

Microbiome research is receiving more attention than in previous years. Scientists are actively investigating how the microbiome may be involved in a wide range of urological diseases [1,2]. Complex microbial communities that inhabit the human body have been acknowledged as important elements closely linked to the pathogenesis of numerous diseases [3,4,5]. Based on conventional urine culture techniques, it was once believed that healthy human urine was a sterile bodily fluid. Slowly or quickly growing bacteria were discovered as distinct commensal flora in the urinary tract with the development of modern DNA sequencing technology, such as 16S ribosomal RNA (rRNA) gene and whole-metagenome sequencing. In 2012, Wolfe et al. used urine collected from adult women without urinary tract infections to perform 16S rRNA gene sequencing, along with traditional urine culture tests, to establish the first evidence of a urinary microbiome [6]. Researchers have put forth numerous hypotheses and reported research findings indicating that the microbiome plays a crucial role in a variety of urological diseases, including overactive bladder syndrome, urolithiasis, and bladder cancer, since the first discovery of the microbiome in the urinary tract [1,7]. These studies have significantly affected how different urological diseases are understood and/or treated. The microbiome and benign prostate hyperplasia or chronic prostatitis are linked, according to numerous detailed studies [8]. The most important question that these studies have not yet resolved is whether or not changes in the microbiome are the root causes of various diseases. There is no doubt that urological diseases and the microbiome are related. Although it is unclear how significant this connection is, there are countless possibilities. Establishing how the microbiome affects urological homeostasis will be crucial to our understanding of urological diseases over the coming years [1]. Bibliometrics is a technique for condensing the statistical analysis of publications in a particular discipline and subject area and for further identifying hot areas of research through the use of infographics [9]. A well-established field of information science called “bibliometric investigation of research fields” allows for the characterization, mapping, and impact evaluation of research fields, journals, authors, and/or articles. The methods have previously been used in surgical specialties, such as urology [10]. The way information is shared in the scientific community has undergone a significant change in recent years. It is more difficult for researchers to access the data they require when there is a wealth of scientific evidence. In order to fully meet the informational needs of researchers and scholars, information retrieval (IR) technologies have been developed. These technologies assist users in finding the most relevant and diverse scientific resources for their questions [11,12,13]. The main goal of this study was to carry out the first characterization of the current research field of the clinical microbiome in urology. First-of-its-kind scientometric insight into the historical development and structural state of the discipline is provided by a field analysis, mapping, and sub-analysis of articles for future research.

## 2. Materials and Methods

Bibliometric techniques were used to analyze the literature on the role of the microbiome in LUTSs. Since the data on the connection that exists between the resident microbiota of the bladder and LUTSs is limited and studies have been focused on other organs’ resident microbiota, such as the gut and the vagina, and their role in LUTSs, we did not consider a limitation to a specific organ microbiome, and contributions made by authors, organizations, and nations/regions as well as the development of theoretical concepts, research sub-themes, and seminal manuscripts in the particular research field were analyzed.

### 2.1. Search Strategy

On 22 December 2022, the entire Scopus database was searched without language or date restrictions. Search terms included “Chronic prostatitis”, OR “Interstitial cystitis”, OR “Lower urinary tract symptoms”, OR “Lower urinary tract dysfunction”, OR “Overactive bladder”, OR “Incontinence”, OR “Urolithiasis”, OR “Urothelium”, OR “Urine”, OR “Urology”, OR “urinary disorder”, OR “Pathophysiology”, OR “Benign prostatic hyperplasia”, OR “Benign prostatic enlargement”, AND “Microbiota”, OR “Microbiome”, OR “Urobioma”, OR “Urobiota; microflora”. Non-English publications, editorials, meeting abstracts and proceedings, letters, errata, retractions, and corrections were excluded using Scopus analysis restriction tools.

### 2.2. Inclusion Criteria

Titles and abstracts of the articles found during the initial search were reviewed. Records that involved a clinical investigation of the microbiome in urology were deemed relevant. Level 1–4 research, systematic reviews and meta-analyses, narrative reviews, and case reports/series were all original articles that were analyzed according to the system presented in Table 1.

**Figure 1:**
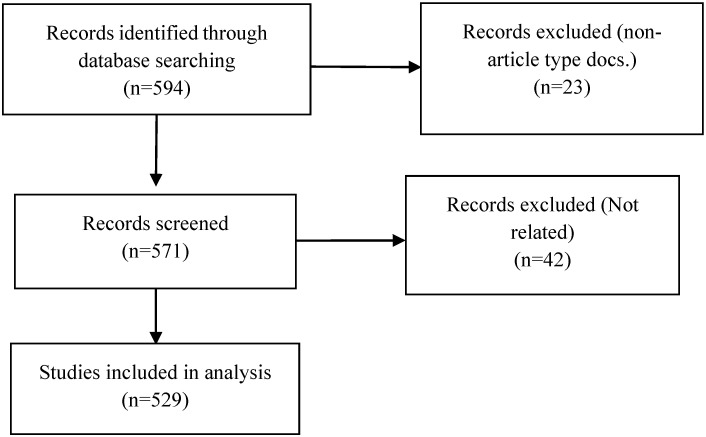
Flow chart for excluding ineligible articles.

Figure 1 presents the inclusion and exclusion process for publications.

### 2.3. Analysis

To record the identified articles and citations, as well as countries, authors, institutions, and journals, Scopus analysis tools were used. In order to rank the main outcomes—country, journal, institution, and author—we quantified research productivity and impact by counting the number of research articles and the number of times each article was cited. The author and institutional data were transformed using the analytical tool Biblioshiny (a Shiny app for Bibliometrix), which took into account variations in author spelling as well as institutional naming and subgroups. The top 10 rankings are broken down by author, institution, and journal productivity. Based on productivity, countries are ranked, and additional continental outputs are summarized. By impact, the top 10 articles in the field are listed.

## 3. Results

The specified search strategy was able to locate 529 documents in total from 267 sources, including journals, books, etc., published from 1981 to 2022. The average number of years from publication was 4.59 years. The average number of citations per document was 21.22, and the average number of citations per year per document was 3.759. The retrieved documents were original articles (*n* = 308), review articles (*n* = 138), notes (*n* = 33), editorials (*n* = 18), letters (*n* = 12), conference papers (*n* = 11), short surveys (*n* = 5), book chapters (*n* = 3), and other types of publications, such as errata (Table 2).

### 3.1. Analysis of Co-Authors

The goal of co-author analysis is to look into the published works of authors and their connections, including the productivity of authors and their institutions, co-authorship networks, and networks of institutions, countries, and/or regions based on the bibliographic records.

### 3.2. Collaboration Network

**Figure 2:**
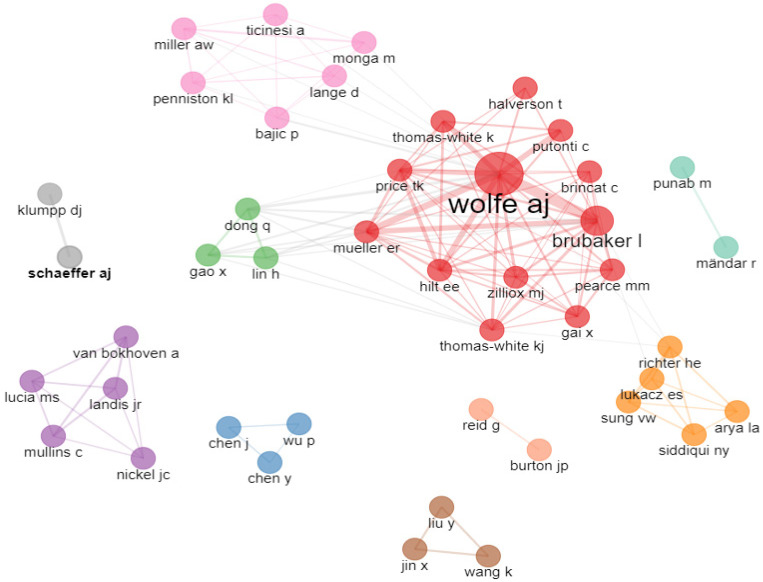
Author collaboration network.

The authors with the most publications were Wolfe AJ and Brubaker I. Figure 2 shows the networks of author collaborations. The size of the nodes indicates the number of publications, while the thickness of the links shows the intensity of the collaborations.

The most productive authors in the field of microbiomes in LUTSs were identified based on the statistical analysis of the chosen journal articles. The authors in this field with the most publications are shown in Figure 2.

**Figure 3:**
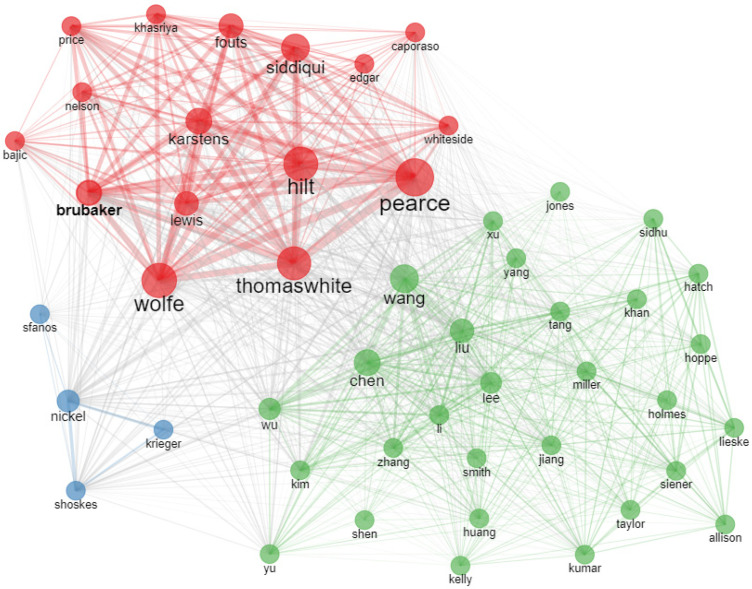
Author co-citation network.

Figure 3 represents the author co-citation network. According to the results, Wolfe, Pearce, Thomas white, and Hilt were the authors with the most co-citations.

### 3.3. Network of Nations, Areas, and Organizations

**Figure 4:**
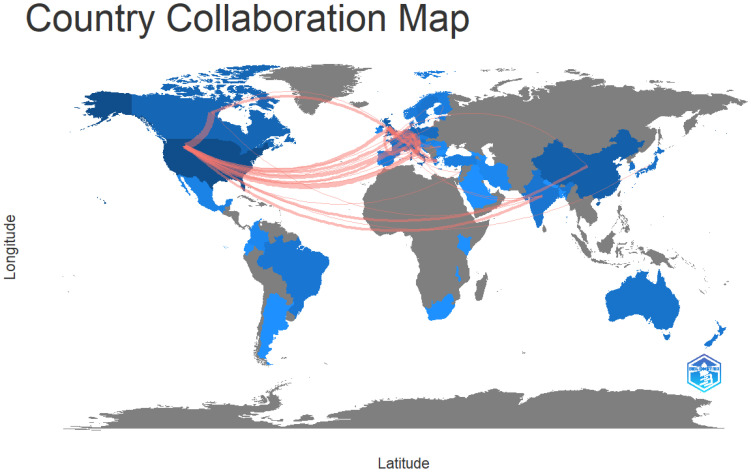
Country collaboration map.

Regarding LUTS-related microbiome research, the three strongest networks were found in the United States, Europe, and China. The country collaboration map is represented in Figure 4.

**Figure 5:**
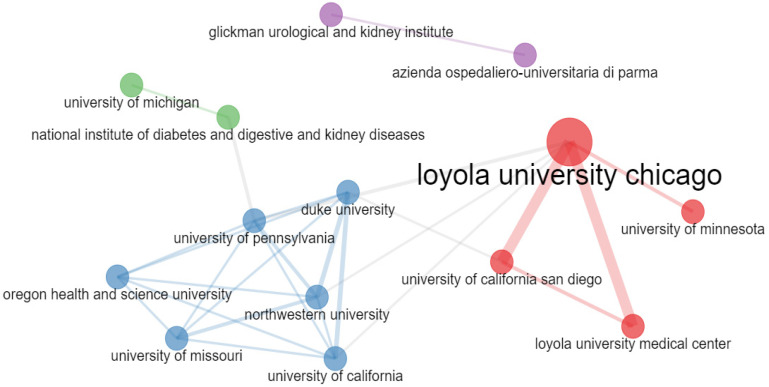
Institutional and/or University Networks.

The top three most collaborative networks were Loyola University Chicago, Loyola University Medical Center, and the University of California San Diego. Figure 5 represents the fourteen most active universities and/or institutes in this field (Figure 5).

### 3.4. Co-Word Evaluation

**Figure 6:**
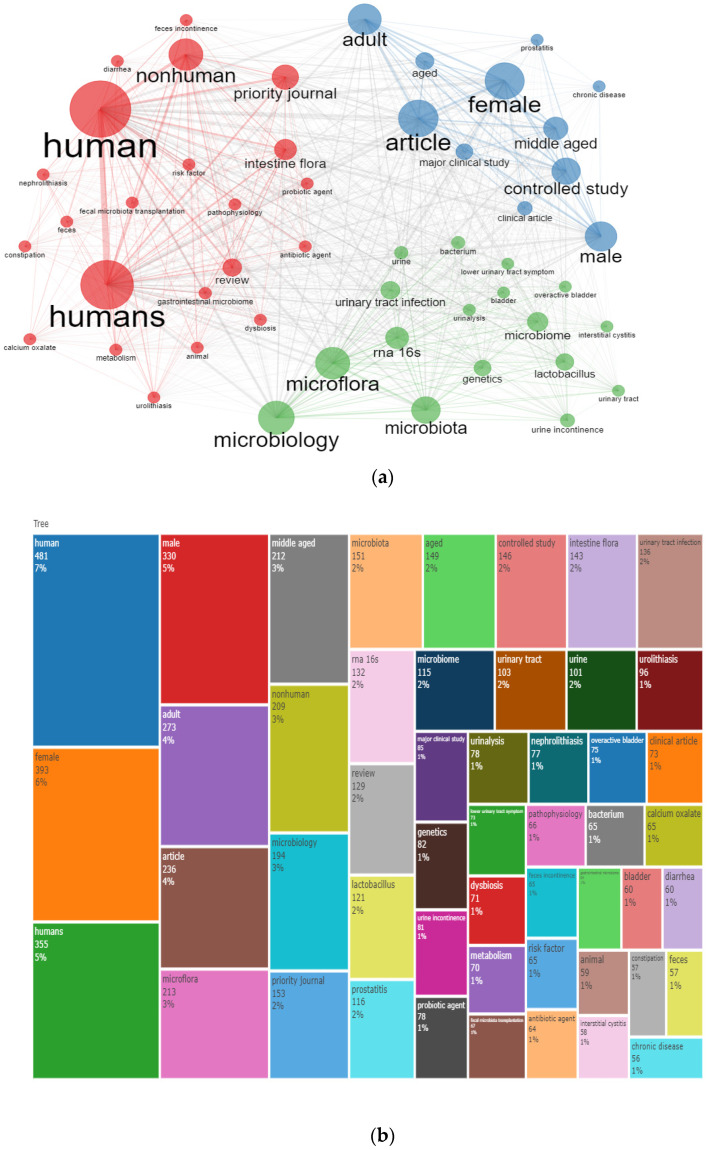
(**a**). Co-Occurring Words Network; (**b**). Keyword treemap.

Co-word analysis shows the co-occurrence of keywords in the researched topics; in addition, it shows the interactions between the searched keywords [14]. The co-occurring words network is presented in Figure 6a, and the keyword treemap is presented in Figure 6b.

The most frequently occurring words among the 50 nodes were: human, humans, nonhuman, female, adult, article, microbiology, microflora, microbiota, controlled study, middle-aged, microbiome, urinary tract infection, intestine flora, RNA 16s, lactobacillus, and genetics.

### 3.5. Co-Citation Evaluation

Author co-citation analysis, document co-citation analysis, and journal co-citation analysis are all subgroups of co-citation analysis. The type of co-citation [15] analysis performed in this study displays the frequency with which two documents were cited jointly in a third article; it also displays the degree of similarity between the two documents. We can understand the direction of the research in a specific time period by understanding the similarities, differences, and relationships between publications [1].

Table 3 presents the most globally cited documents. The most cited article was by Paramsothy S, published in the journal *Lancet* in 2017, with a total of 684 total citations.

**Figure 7:**
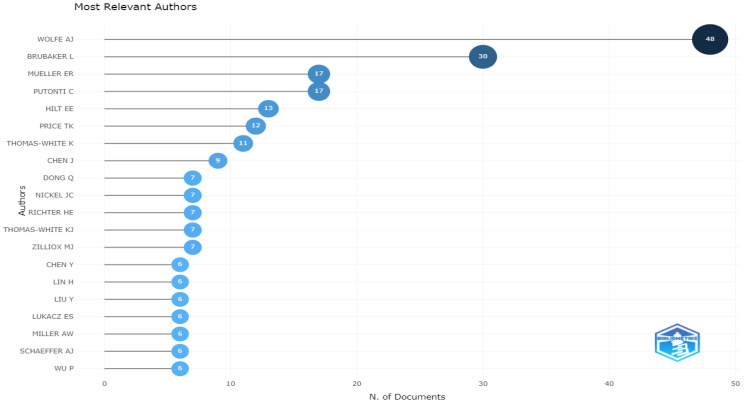
Most locally cited authors.

The most locally cited authors are presented in Figure 7. According to the results, Wolfe AJ and Brubaker L were the most locally cited authors, with a total of 48 and 30 citations, respectively.

**Figure 8:**
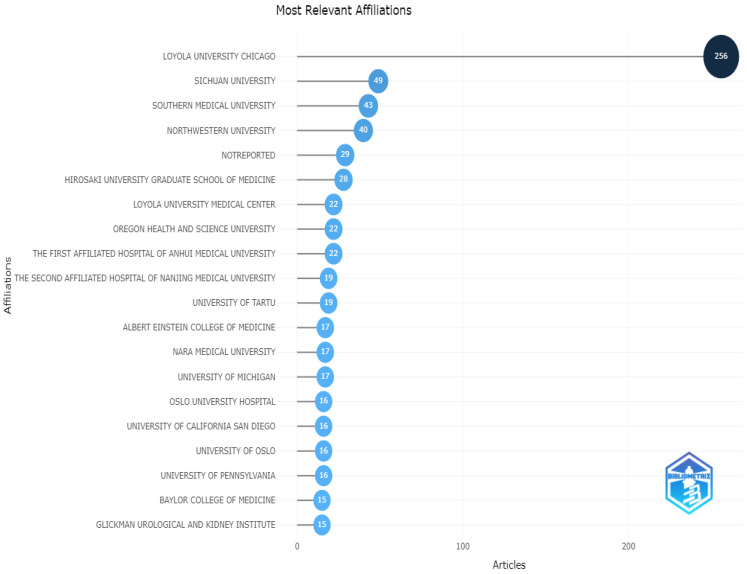
Most relevant affiliations.

The most relevant affiliations are presented in Figure 8. According to the analysis, Loyola University Chicago was in the first rank, and Sichuan University and Southern Medical University were the other top universities in this field.

**Figure 9:**
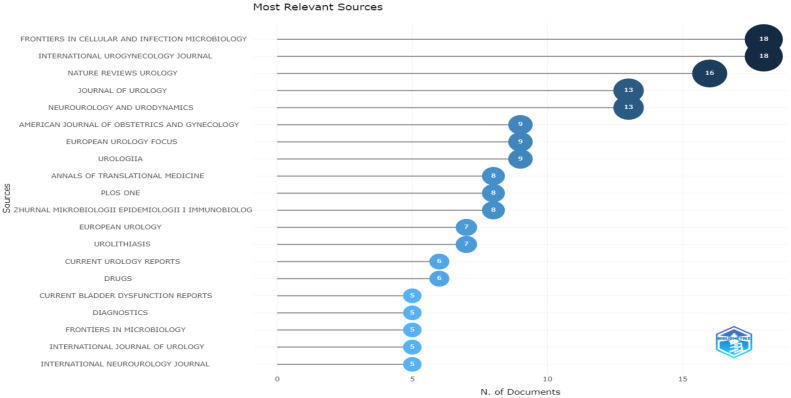
Most relevant sources.

*Frontiers in Cellular and Infection Microbiology* and the *International Urogynecology Journal*, followed by *Nature Reviews Urology*, were the top three most relevant sources in microbiome research in LUTSs, with 18, 18, and 16 published articles, respectively (Figure 9).

**Figure 10:**
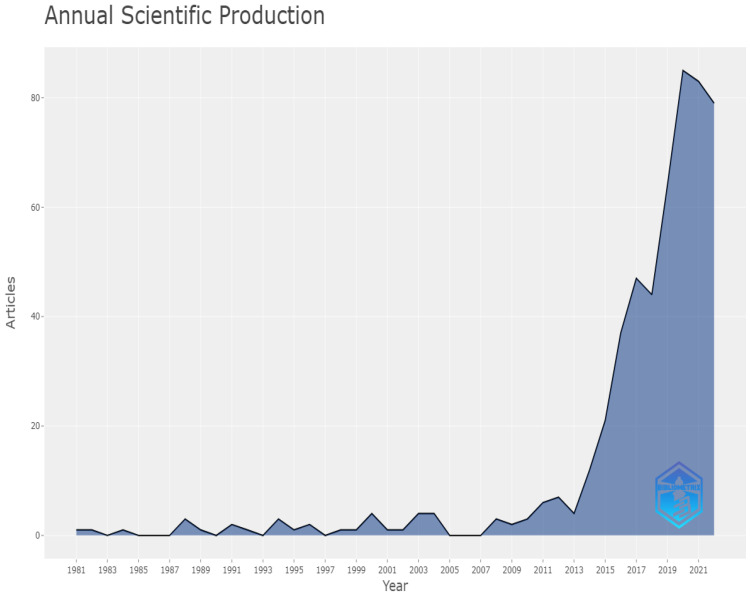
Annual rate of scientific production.

Figure 10 depicts the annual rate of scientific production, with the oldest article published in 1981 and the newest published in 2021.

**Figure 11:**
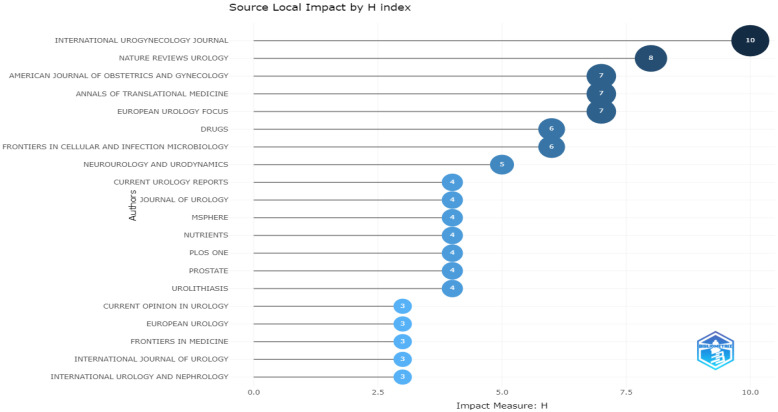
Source local impacts according to h-indexes.

The highest source local impact according to the h-index was achieved by the *International Urogynecology Journal*, followed by *Nature Reviews Urology* (Figure 11).

In Table 4, source impact data, including h-indexes, g-indexes, m-indexes, total citations, and publication start dates, are presented.

**Figure 12:**
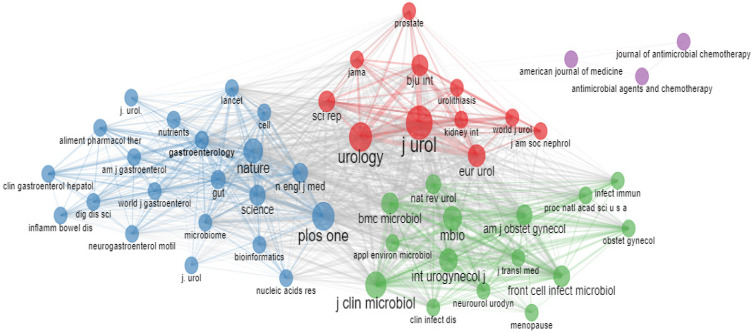
Co-citation network.

Figure 12 depicts the source co-citation network. The most prominent journals in this field, according to the scores, among 50 nodes, were the *Journal of Urology*, *Urology*, *Nature*, *PLOS One*, the *Journal of Clinical Microbiology*, the *International Urogynecology Journal*, and *mBio*.

In 2002, Yang MH’s article was the only one to receive a co-citation; however, after 2006, a citation network emerged and grew rapidly over time.

## 4. Discussion

As the volume of microbiome research in urological diseases expands, it becomes increasingly important for healthcare providers, trainees, and research groups to identify the most relevant literature and stakeholders. Researchers are actively investigating the role of the microbiome in a wide range of urological illnesses, and microbiome research is currently receiving increased attention [16]. Complex microbial communities that inhabit the human body are now understood to play a significant role in the pathophysiology of many diseases. Systematic analysis of all relevant papers and materials on this topic utilizing bibliometric analysis can be more visually appealing than standard reviews. Researchers who are new to the topic will find this method useful because it can display visual results, enabling them to better understand the integral tendencies of the field under investigation. Additionally, it can highlight current research hotspots, emerging future trends, and ground-breaking papers. It was discovered that numerous different research papers on the microbiome and LUTSs have been published over the course of the study, and the number of publications is rising daily. Analyzing these publications in this context can be beneficial and applicable in illuminating the tendencies and characteristics with respect to subjects, authors, journals, nations, and activities in this area of medicine, as well as the attempts to publish scientific evidence.

Even though the terms “microbiome” and “microbiota” are frequently used interchangeably today, some authors believe that the term “microbiome” actually refers to the collective genome of the microbial population. Despite having been called out, this terminological uncertainty still exists [17,18]. Compared to “microbiota,” the term “microbiome” is relatively new. It is possible that there was some ambiguity in the concept’s early meanings because it was still developing at the time. To shed light on the categorization and topical dynamics that have been observed, we add some remarks about the beginning and development of both terms. Basically, the term “biota”, as it was first defined by the zoologist Leonhard Stejneger [19], might be seen as connected to species richness in terms of flora and fauna composition. Microbiota-related studies initially developed in accordance with this primary definition of “biota”. While identifying species in macro-communities is a relatively simple task based on vast prior expertise, when dealing with the inhabitants of anaerobic environments, it is more difficult. As the bulk of microbial species have turned out to be unculturable, 1960s-era culture techniques have reached their limits. The discovery made in the 1990s that metagenomic methodologies could be used to investigate environmental samples, primarily from aquatic and soil ecosystems, allowed for the examination of complicated species combinations [20]. Following this, a more in-depth examination of the microbial communities that live inside humans was motivated by the availability of new tools as well as a paradigm shift toward the ecological notions of biodiversity, equilibrium, and host–microbiome–environment interactions. The term “biome” refers to “an association of various, mutually dependent organisms in a natural ecological unit” and implies a certain structure, dynamics, and set of functional linkages. Thus, the idea of the “biome” and functional metagenomics marked a turning point in the study of the intricate interactions between the host, its environment, and its symbionts. In our study, this conceptual shift has been tracked as the field and study topic have developed. Due to this significant change, the field of microbiota research has been able to expand by bridging disciplinary lines and produce cutting-edge therapeutic applications. It needs to be seen whether the published corpus updated with publications containing the phrase “microbiome” reveals the various patterns and subjects. A more thorough presentation of microbiome research using more exhaustive search queries could be a challenge for future studies [17,21].

The most productive nations/regions were found to be the USA, China, and Europe, and the USA had the most publications. Between emerging and developed countries, there was considerable inequity. It is anticipated that developing nations will keep investing in research.

In the years considered, the active researchers that were identified via our search of the Scopus database used urine from adult women without urinary tract infections to perform 16S rRNA gene sequencing, together with traditional urine culture techniques, to establish the first evidence of a urinary microbiome. Researchers have put forward numerous hypotheses and documented study findings indicating that the microbiome plays a crucial role in a variety of urological illnesses, including overactive bladder, urolithiasis, and bladder cancer, since the initial discovery of the microbiome in the urinary tract. A very intriguing finding is the observation that urological disorders, as opposed to infectious diseases, are associated with the microbiome [6].

Publications with significant citation bursts indicate that the researchers closely followed these papers throughout time. We specifically divided the publications into review and original research types for the top citation references. Research publications with high citation counts involve numerous experiments and contributors [22].

Another study [23] reviewed the established linkages between the gut microbiota and the development of renal stones and stated that bacteria are utilized to stop the recurrence of bladder cancer. In addition, the authors emphasized that urologists will need to take the microbiome’s potential effects on the diagnosis and treatment of specific urological disorders into account in the future. Finally, they came to the conclusion that fresh perspectives might make it possible to anticipate the propensity to acquire specific urological disorders as well as create novel therapeutic approaches. According to our search, two top-cited articles were original. One of them showed that many of the organisms identified in urine by 16S rRNA gene sequencing are, in fact, cultivable using an expanded quantitative urine culture (EQUC) protocol via a modified culture method that includes plating larger volumes of urine, incubation under different atmospheric conditions, and prolonged incubation times. Using both conventional and EQUC culture techniques, 65 urine samples (from 24 controls and 41 patients with overactive bladders) were studied. While the majority of these (48/52 (92%) of the 65 urine samples) were reported by the clinical microbiology laboratory using the usual urine culture methodology as exhibiting no growth at 10(3) CFU/mL, 52 of the 65 urine samples (80%) developed bacterial species utilizing EQUC. EQUC enabled the identification of 35 distinct genera and 85 distinct species. Following Lactobacillus (15%), in terms of frequency of isolation, were Corynebacterium (14.2%), Streptococcus (11.9%), Actinomyces (6.9%), and Staphylococcus (6.9%). Other frequently isolated genera include Actinobaculum, Gardnerella, Aerococcus, and Bifidobacterium [24]. The other top-cited paper [25] investigated the microbiome of urine collected by transurethral catheter from women seeking therapy for urgency urinary incontinence (UUI), and a control group of women without UUI was characterized using both 16S rRNA gene sequencing and EQUC. The authors found statistically significant changes in the frequency and abundance of bacteria using both methods, pointing to a potential function for the urine microbiome in the health of female urinary systems.

Few publications appear in many new domains, showing the nascency of interest, as microbiota research is still concentrated in the several fields in which it historically developed. Due to technical advancements, researchers in numerous domains become familiar with a topic during the expansion phase and discover new applications. The growing understanding of how the microbiome affects human health attracts a lot of funding, which encourages the publication of fresh findings [26]. In the current study, Loyola University Chicago was the affiliation that was most prominent. There is a transdisciplinary translational research team made up of physicians, clinical microbiologists, fundamental scientists, and bioinformaticians called the Loyola Urinary Education and Research Collaborative (LUEREC). Members of LUEREC collaborate to comprehend how genitourinary wellness and disease are impacted by communities of bacteria (microbiota) that live in the bladder and other niches of the genitourinary tract. By demonstrating through the use of DNA and culture data that the “healthy” bladder is home to its own distinct microbiota, LUEREC has disproven the conventional wisdom that urine is sterile. While research into the urobiome, also known as the urinary microbiota, is still in its early stages, its characterization could help with the diagnosis and treatment of genitourinary-related conditions, such as urgency urinary incontinence, urinary tract infections, urothelial cell carcinoma, and urinary stone disease.

A study field’s hot spots and trends are indicated by keywords. Article keywords can provide essential details about the subject or the central thesis of a particular study [27]. The high-frequency keywords microbiology, microflora, microbiota, microbiome, urinary tract infection, gut flora, RNA 16s, lactobacillus, genetics, etc., were all given a typical overview by keyword co-occurrence analysis.

The goal of scientometric evaluations of disciplines is to encourage additional research and collaboration by facilitating knowledge acquisition and progress. Research teams should take note of the best publications and stakeholder works when conceptualizing, keeping important field themes in mind. Since all of the citations in this field were found based on the selected keywords, we did not focus on innovations or discovery studies; therefore, it is one of the limitations of the present research that original work conducted by centers with a focus on discovery or innovation may not have been retrieved. Burst keywords indicate major research directions over the past ten years and are suitable research topics for field research teams [28]. The most recent emerging fields, e.g., the role of the microbiome in urological diseases or lower urinary tract dysfunction, overactive bladder syndrome, chronic prostatitis, interstitial cystitis, incontinence, prostate cancer, and urolithiasis, may be preferable options, as it may become more challenging to continue innovation in each area over time.

The single Scopus search and category restriction were limitations of this study; however, prior research has shown that studies restricted to the Scopus database seem to collect datasets that correctly reflect fields because of the high percentage of indexed high-impact-factor journals. Scopus is the largest abstract and citation database of peer-reviewed literature—scientific journals, books, and conference proceedings. Delivering a comprehensive overview of the world’s research output in the fields of science, technology, medicine, social sciences, and arts and humanities, Scopus features smart tools to track, analyze, and visualize research [29]. However, the limit of the analysis to journals indexed in the database may have meant that some relevant papers were missed.

Language and article-type restrictions were imposed: non-English articles, as well as editorials, comments, and conference proceedings, which influence citation rates, were all excluded.

Due to the inherent bias in the literature, these literary forms are not well liked or frequently quoted; thus, there was little chance that their omission would have considerably affected the outcome. Only a few of those were used in this analysis, including the citation metrics. Rankings may differ if using different metrics, because the citation metrics employed in this study were just a few of those frequently used in the literature. Articles about the microbiome in LUTS research were primarily published in microbiology and urology journals. The other top 5 journals in the subset were all urological, which was reflected in the top 10 rankings for the field (6 of the top 10 journals represented urology). Overall, the top journals reflected the medical/surgical specialties of the area.

According to our results based on the search strategy and single-database search, most of the mentioned studies were epidemiolocal, and it seems that the field is open for other study designs, such as diagnostic and randomized controlled trials. Regarding the newness of the subject, the roadmap for future research should be determined.

## 5. Conclusions

One of the most crucial requirements for developing research policies and anticipating the scientific requirements of researchers is observing the evolution of various scientific fields. Understanding research gaps and future needs in microbiome research in LUTSs can be effectively understood by paying attention to the models, maps, and visualizations used in this research, which are the results of systematic analysis of scientific products in the most esteemed scientific journals in the world. Although results in other urological fields are comparable, Level-1 evidence is typically lacking in the top-ranked literature. Future bibliometric investigations of the field would be served by analysis of the role of the microbiome in urological disease subsets to further guide output.

## Figures and Tables

**Table 1:** Levels of evidence.

Level of Evidence	Type of Study
1a	Systematic review of (homogeneous) randomized controlled trials
1b	Individual randomized controlled trials (with narrow confidence intervals)
2a	Systematic review of (homogeneous) cohort studies of “exposed” and “unexposed” subjects
2b	Individual cohort study/low-quality randomized controlled studies
3a	Systematic review of (homogeneous) case–control studies
3b	Individual case–control studies
4	Case series, low-quality cohort or case–control studies

**Table 2:** Main scientometric information.

Description	Results
MAIN INFORMATION ABOUT DATA	
Timespan	1981:2022
Sources (journals, books, etc.)	267
Documents	598
Average number of years from publication	4.59
Average citations per documents	21.22
Average citations per year per document	3.759
References	24,140
DOCUMENT TYPES	
Article	377
Book chapter	3
Conference paper	11
Editorial	18
Erratum	1
Letter	12
Note	33
Review	138
Short survey	5
DOCUMENT CONTENTS	
Keywords plus (ID)	4847
Author’s keywords (DE)	975
AUTHORS	
Authors	2279
Author appearances	3032
Authors of single-authored documents	55
Authors of multi-authored documents	2224
AUTHOR COLLABORATION	
Single-authored documents	66
Documents per author	0.232
Authors per document	4.31
Co-authors per document	5.73
Collaboration index	4.8

**Table 3:** Most globally cited documents.

Paper	DOI	Total Citations	TCs per Year	Normalized TCs
CANI PD, 2018, GUT	10.1136/gutjnl-2018-316723	632	126.4	16.6515
HILT EE, 2014, J CLIN MICROBIOL	10.1128/JCM.02876-13	415	46.1111	6.0364
PEARCE MM, 2014, MBIO	10.1128/mBio.01283-14	358	39.7778	5.2073
WHITESIDE SA, 2015, NAT REV UROL	10.1038/nrurol.2014.361	318	39.75	5.9839
CAMPOLI-RICHARDS DM, 1988, DRUGS	10.2165/00003495-198835040-00003	313	8.9429	2.4646
DONSKEY CJ, 2004, CLIN INFECT DIS	10.1086/422002	309	16.2632	3.9615
SFANOS KS, 2018, NAT REV UROL	10.1038/nrurol.2017.167	198	39.6	5.2168
CAMPIERI C, 2001, KIDNEY INT	10.1046/j.1523-1755.2001.0600031097.x	192	8.7273	1
PEARCE MM, 2015, AM J OBSTET GYNECOL	10.1016/j.ajog.2015.07.009	166	20.75	3.1237
MING X, 2012, J PROTEOME RES	10.1021/pr300910n	165	15	3.319
THOMAS-WHITE KJ, 2016, INT UROGYNECOL J	10.1007/s00192-015-2847-x	142	20.2857	5.2279
WEST NP, 2011, NUTR J	10.1186/1475-2891-10-30	139	11.5833	4.9349
SON JS, 2015, PLOS ONE	10.1371/journal.pone.0137725	137	17.125	2.578
KARSTENS L, 2016, FRONT CELL INFECT MICROBIOL	10.3389/fcimb.2016.00078	136	19.4286	5.007
ROSS JJ, 2003, MEDICINE	10.1097/01.md.0000091180.93122.1c	136	6.8	2.2204
PEYRONNET B, 2019, EUR UROL	10.1016/j.eururo.2019.02.038	130	32.5	7.7108
SIDDIQUI H, 2012, BMC MICROBIOL	10.1186/1471-2180-12-205	126	11.4545	2.5345
ARAGÓN IM, 2018, EUR UROL FOCUS	10.1016/j.euf.2016.11.001	121	24.2	3.188
HOTA SS, 2017, CLIN INFECT DIS	10.1093/cid/ciw731	117	19.5	3.0148

**Table 4:** Source Impacts.

Element	h_Index	g_Index	m_Index	TC	NP	PY_Start
ACR OPEN RHEUMATOLOGY	1	1	1	2	1	2022
ADVANCES IN EXPERIMENTAL MEDICINE AND BIOLOGY	1	1	0.043478261	108	1	2000
ADVANCES IN UROLOGY	1	1	0.5	2	1	2021
AGE AND AGEING	2	2	0.2	34	2	2013
AGING	1	1	0.5	7	1	2021
AKUSHERSTVO I GINEKOLOGIYA (RUSSIAN FEDERATION)	1	1	0.25	2	1	2019
AMERICAN JOURNAL OF GASTROENTEROLOGY	1	1	0.111111111	10	1	2014
AMERICAN JOURNAL OF INFECTION CONTROL	1	1	0.333333333	2	1	2020
AMERICAN JOURNAL OF KIDNEY DISEASES	1	1	0.25	20	1	2019
AMERICAN JOURNAL OF OBSTETRICS AND GYNECOLOGY	7	9	0.28	466	9	1998
AMERICAN JOURNAL OF PHYSIOLOGY-REGULATORY INTEGRATIVE AND COMPARATIVE PHYSIOLOGY	1	1	0.5	2	1	2021
AMERICAN JOURNAL OF REPRODUCTIVE IMMUNOLOGY	1	1	0.25	41	1	2019
AMERICAN JOURNAL OF TRANSPLANTATION	1	1	0.166666667	24	1	2017
ANAEROBE	1	2	0.05	24	2	2003
ANDROLOGIA	2	2	0.1	99	2	2003
ANDROLOGIA I GENITAL’NAA HIRURGIA	1	1	0.333333333	1	1	2020
ANDROLOGY	1	1	0.333333333	9	1	2020
ANNALS OF CLINICAL MICROBIOLOGY AND ANTIMICROBIALS	2	2	1	8	2	2021
ANNALS OF GASTROENTEROLOGY	1	1	0.5	5	1	2021
ANNALS OF THE ROYAL COLLEGE OF SURGEONS OF ENGLAND	1	1	0.111111111	1	1	2014

## Data Availability

Not applicable.
